# Empowering Vision Transformer by Network Hyper-Parameter Selection for Whole Pelvis Prostate Planning Target Volume Auto-Segmentation

**DOI:** 10.3390/cancers15235507

**Published:** 2023-11-21

**Authors:** Hyeonjeong Cho, Jae Sung Lee, Jin Sung Kim, Woong Sub Koom, Hojin Kim

**Affiliations:** 1 Department of Radiation Oncology, Yonsei Cancer Center, Heavy Ion Therapy Research Institute, Yonsei University College of Medicine, Seoul 03722, Republic of Korea; hyjcho12@gmail.com (H.C.); jinsung@yuhs.ac (J.S.K.); 2 Department of Biomedical Sciences, Seoul National University College of Medicine, Seoul 03080, Republic of Korea; jaes@snu.ac.kr

**Keywords:** transformer, hyper-parameter selection, planning target volume, auto-segmentation, prostate cancer, VT U-Net v.2

## Abstract

**Simple Summary:**

Vision transformers have been recently spread out to enhance segmentation accuracy, becoming an active area of research and development involved in radiotherapy. We found that the new network architecture did not guarantee improvement. Conventional CNN-based networks struggled with being expanded to the auto-segmentation of tumors from normal organs due to local geometric dependence and difficulty in the hyper-parameter selection. As seen in the development and success of nnU-Net, we emphasized the importance of finding suitable hyper-parameters for the vision transformer. We applied our proposed framework based on VT U-Net v.2 to the prostate target volume segmentation, followed by extensively validating its performance in segmentation accuracy against the other five competing deep neural networks through four-fold cross-validation using CT images.

**Abstract:**

U-Net, based on a deep convolutional network (CNN), has been clinically used to auto-segment normal organs, while still being limited to the planning target volume (PTV) segmentation. This work aims to address the problems in two aspects: 1) apply one of the newest network architectures such as vision transformers other than the CNN-based networks, and 2) find an appropriate combination of network hyper-parameters with reference to recently proposed nnU-Net (“no-new-Net”). VT U-Net was adopted for auto-segmenting the whole pelvis prostate PTV as it consisted of fully transformer architecture. The upgraded version (v.2) applied the nnU-Net-like hyper-parameter optimizations, which did not fully cover the transformer-oriented hyper-parameters. Thus, we tried to find a suitable combination of two key hyper-parameters (patch size and embedded dimension) for 140 CT scans throughout 4-fold cross validation. The VT U-Net v.2 with hyper-parameter tuning yielded the highest dice similarity coefficient (DSC) of 82.5 and the lowest 95% Haussdorff distance (HD95) of 3.5 on average among the seven recently proposed deep learning networks. Importantly, the nnU-Net with hyper-parameter optimization achieved competitive performance, although this was based on the convolution layers. The network hyper-parameter tuning was demonstrated to be necessary even for the newly developed architecture of vision transformers.

## 1. Introduction

Segmentation of tumors and normal organs is a crucial procedure in radiotherapy (RT) treatment planning because it shows the amount of radiation delivered to the target volume and the organs delineated in the optimized plan. However, this segmentation is often time-consuming and labor-intensive, and requires a steep learning curve to reach the expert level. Furthermore, despite several automated methods proposed over the past few decades, their segmentation accuracy has been inconsistent, primarily due to their reliance on a limited number of patient cases [1,2,3,4]. Fortunately, the advent of deep neural networks empowered by advanced computing technology, particularly graphical processing units (GPUs), has opened up new possibilities for medical image segmentation [5]. This utilization of deep neural networks has led to a learning-based approach, in which algorithmic development and assessment are performed using a significant amount of data with a division of training and testing phases [6]. Additionally, convolutional neural networks (CNNs) incorporate convolution operators into deep neural networks, thus enabling 2D or 3D images to be directly applied for training the networks [7,8].

U-Net has succeeded in various CNN applications, particularly in medical image processing, using the convolutional layers and the skip connections between the encoder and decoder [9]. Among these applications, the normal organ segmentations in RT have been the most active area of research and development [10,11], which have resulted in the current availability of several software options for auto-segmentation. However, CNN-based frameworks have yet to be widely expanded to tumor segmentation as they mainly focus on segmenting normal organs in most cases [12]. One of the reasons for this limitation is the inherent challenges and variations in tumor segmentation. Unlike normal organs, tumors in medical images lack clear gradients or typical characteristics regarding shape, size, or location. Another reason is the shortcomings of CNN-based architectures, including the difficulty in optimizing network hyper-parameters and the excessive dependence on local imaging information. For example, there are hyper-parameters such as image size, learning rate, the depth of the network, and the kernel size of CNN that need to be determined by users, which can affect the segmentation accuracy. Additionally, the convolutional layers of CNN in the encoders and decoders extract and propagate the image features using a small kernel matrix, typically 3 × 3, which limits the exploration of the global spatial information of the images.

Several studies have addressed the drawbacks of CNN-based models for medical image segmentation. Although several U-Net variants have been developed to enhance segmentation accuracy, several studies by Isensee et al. in 2018 and 2021 showed that a well-trained U-Net was still the most effective approach for achieving greater segmentation accuracy [13,14]. Furthermore, their studies confirmed that the qualified segmentation accuracy was attained by completing the well-trained U-Net by the hyper-parameter optimization on nnU-Net (“no-new-Net”). Meanwhile, a new type of network architecture, the transformer, has been actively developed in the context of deep learning. Transformers extract global and remote semantic information, crucial for dense prediction tasks, especially for 3D medical image segmentation [15,16]. Unlike CNNs, transformers suppress irrelevant areas of the input image and highlight salient features useful for a specific task [17]. The combination of CNN and transformer has been studied in the encoders of 2D and 3D networks, such as TransUNet and Unetr [18,19]. In TransUNet, CNN feature maps were fed into the encoder of the transformer. Contrarily in Unetr, the features extracted from the transformer entered CNN layers. Recently, fully transformers with U-Net shapes have been introduced in 2D or 3D image-based networks: Swin-Unet [16], nnFormer (almost fully) [20], and VT U-Net [21]. These fully transformers have the characteristics of a hierarchically shifted window in the U-shaped networks.

This study aims to enhance the precision of auto-segmentation of the whole pelvis target volume for prostate cancer patients, which contains extensive lymph nodes and lacks a clear gradient in image intensity. To achieve this goal, the study focused on using a new network architecture called a vision transformer, which can overcome the defects of CNN-based networks. Furthermore, the study aims to identify the suitable network hyper-parameters that may impact the PTV segmentation accuracy. The VT U-Net v.2 was selected for the whole pelvis planning target volume (PTV) segmentation for prostate cancer patients as it was featured in (1) a U-shaped transformer network architecture consisting of fully self-attention blocks and (2) a function of semi-hyper-parameter selection for the transformer based on nnU-Net. Since the VT U-Net v.2 partially accounted for some network hyper-parameters necessary for the vision transformer, the proposed study exploited additional hyper-parameter tuning and a newly defined loss function to address this limitation.

Our main contributions of this work were as follows:

- We propose one of the initial auto-segmentation models for the PTV target volume of the prostate, as the previous investigators have predominantly focused on studying organs-at-risk (OARs) and extended it to gross tumor volume/clinical target volume (GTV/CTV) to some extent.
- We demonstrated that the proposed model outperforms the latest state-of-the-art (SOTA) models in the PTV auto-segmentation, including nnU-net and recently proposed hybrid- and fully-vision transformers.

We validated the effectiveness of fine-tuning the important network hyper-parameters in the new network architecture, vision transformer, for enhancing the PTV segmentation accuracy.

## 2. Materials and Methods

### 2.1. Transformer VT U-Net

**Figure 1:**
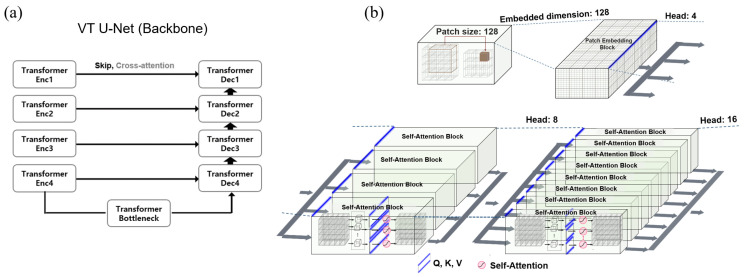
(**a**) VT U-Net architecture, (**b**) primary hyper-parameters (patch size, embedded dimension, and head numbers) in VT U-Net v.2.

This work adopted the fully vision transformer for prostate target volume auto-segmentation, specifically the VT U-Net featuring self-attention without convolution layers in a U-shaped encoder and decoder, as seen in Figure 1a. Self-attention is a crucial component of the transformer, enabling the representation of the degree of impact as a correlation by shifting a single sequence to different sequences, thus handling the global receptive field intrinsically [22,23,24,25]. Furthermore, instead of updating the convolution filters as typically performed in a CNN [26], the self-attention mechanism updates three matrices in parallel, namely query (Q), key (K), and value (V) vectors.

Among the hyper-parameters required for the vision transformer, the embedded dimension is the number of channel dimensions for linearly projecting input data into the first feature map. The patch size represents the size of volumetric data for partitioning during training. The batch size is an additional dimension fixed across the epoch, representing the number of input data. The window size means the size of the data operated in self-attention, while the head number referrs to the number of self-attention units. As shown with a blue line in Figure 1b, the embedded dimension was uniformly split by the head number, thus determining the sizes of Q, K, and V vectors feeding into the self-attention mechanism. The attention map ($R^{N\times N}$) showed how much attention was given to the entire image area to identify which features contributed the most, with N representing the cube of the window size. To set the head numbers, we based it on the embedded dimension of the pre-trained Swin-transformer model [27,28], with [3, 6, 12, 24] set to 96, [4, 8, 16, 32] set to 128, and [6, 12, 24, 48] set to 192.

### 2.2. Hyper-Parameter Selections for Network Architecture of VT U-Net

The new vision transformer network architecture with self-attention can help address the issue of relying on local geometric imaging information during network training. However, optimal network performance can only be guaranteed by carefully selecting network hyper-parameters and properly considering architectural details. The nnU-Net demonstrates the importance of hyper-parameter optimization over the development of the novel network architecture. The basic concept of nnU-Net is to ensure computational efficiency by reflecting the GPU memory budget through their statistics, which helps determine essential network hyper-parameters such as the patch size of input images, batch size, and the number of convolution layers. Additionally, nnU-Net applies the post-processing to mainly remove noisy elements and proposes a novel concept of defining the loss function, called deep supervision, serving as an overall objective function across the output layers of different resolutions.

While VT U-Net v.1 does not prioritize hyper-parameter optimizations, it emphasizes the new network architecture. VT U-Net v.2 was an upgraded version incorporating an adaptive hyper-parameter optimizer embedded in nnU-Net, rather than revising the network architectures. Table 1 summarizes the features of VT U-Net v.1 and VT U-Net v.2, compared to nnU-Net. VT U-Net v.2 adopted useful features from nnU-Net to improve the performance, mainly oriented to GPU memory efficiency, which did not pay much attention to optimizing the hyper-parameters that potentially affect the network architecture. It is worth noting that nnU-Net was based on convolution layers and did not cover up the hyper-parameters associated with the vision transformers. Likewise, VT U-Net v.2, referring to nnU-Net, did not fully encompass the necessary network hyper-parameters for the vision transformers, such as embedded dimension, head numbers, and window size, as outlined in Table 1. Additionally, it did not provide specific guidelines for the patch size and the number of layers.

**Figure 2:**
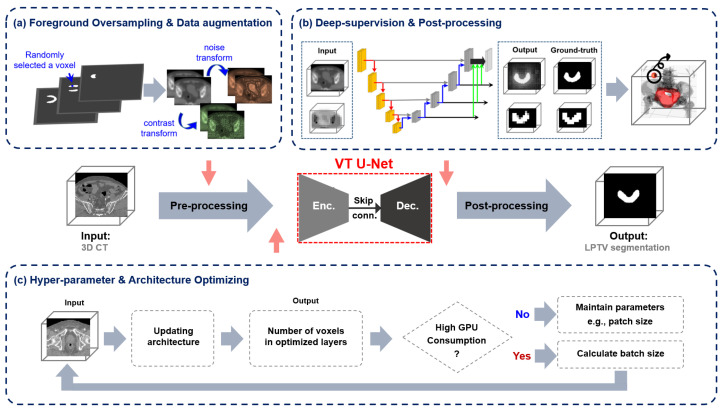
The main framework of the proposed network based on VT U-Net v.2 for auto-segmentation of the whole pelvis prostate planning target volume (PTV): (**a**) adaptive workflow in pre-processing, (**b**) deep supervision and post-processing, and (**c**) hyper-parameters and architecture selection considering graphical processing units (GPU) memory efficiency.

The same approach as nnU-Net was employed in this study to determine the number of layers based on the depth of the network, and a window size of 7, consistent with previous transformer-based networks, was set. Based on the pre-trained models, the embedded dimension and head numbers were adjusted accordingly. The embedded dimension and patch size play a vital role in determining the dimensional specifications of the first feature input into the network. Of the two, the patch size is crucial since it directly influences the trade-off between global and local information processing in the network. Larger patch sizes capture more global context for recognizing larger-scale patterns and structures in the image, while smaller ones catch more local fine-grained details. Therefore, the first feature extracted from various patch sizes included other contextual information that may affect the performance of the transformer. Also, the VT U-Net v.2 did not have deep supervision in defining the loss function. To address this, the modified VT U-Net v.2 added an auxiliary segmentation output to depthwise layers by applying a 1 × 1 × 1 convolution to enable this deep supervision to alleviate the vanishing gradient issue by effectively utilizing the multi-level loss fusion [29,30,31,32]. Figure 2 illustrates the proposed network architecture, including pre-processing the given input images, post-processing the generated output, the structure of the deep supervision, and the hyper-parameter selection. Table 1 outlines the differences between VT U-Net v.2 and our proposed framework, mainly regarding hyper-parameter selections.

**Figure 3:**
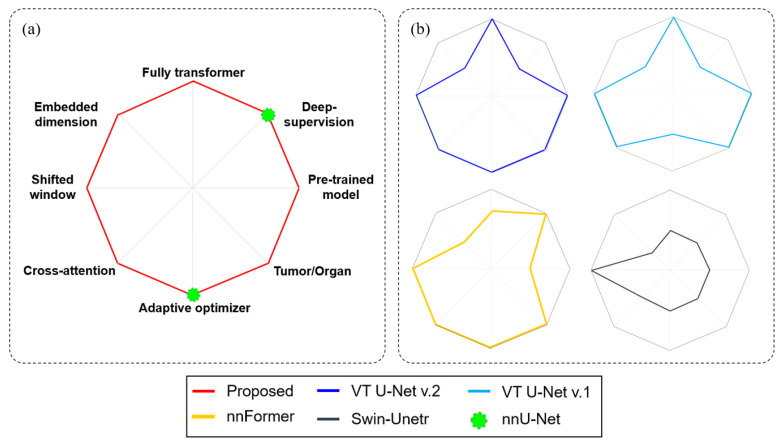
Diagram of comparison with transformer-based networks (**b**) and the proposed network (**a**). All diagram components were classified into binary (yes or no).

Figure 3 elaborates the competence of the proposed framework compared to the characteristics of several other transformer-based networks, such as Swin-Unetr, nnFormer, VT U-Net v.1, and VT U-Net v.2, and some specific features of nnU-Net. As stated above, the proposed network, called modified VT U-Net v.2, was based upon the VT U-Net v.2, and included deep supervision in defining the loss function and intensified the degree of care for hyper-parameters. The proposed network differed from nnFormer and Swin-Unetr in terms of the network architecture (fully transformer vs. a combination of transformer and convolution layers) and usage of the pre-trained model. Furthermore, the proposed network selected the higher embedded dimension than those in the other investigated networks for tumor segmentation. Finally, while VT U-Net v.2 partially used an adaptive optimizer oriented from nnU-Net, the proposed network handled it more comprehensively, as shown in Table 1.

### 2.3. Patient Cohorts and Data Pre-Processing

This study protocol was approved by the ethics committee/institutional review board (IRB) of the Yonsei University Severance Hospital, Korea (4-2022-0894), which waived the need for informed patient consent to the use of patient images. The patient cohort consisted of 160 cancer patients who received RT from 2019 to 2020 after being diagnosed with prostate cancer spread in the whole pelvis [33,34], which one radiation oncologist retrospectively observed. All data used in this work were acquired from a single institution, and the target volume was delineated by an experienced radiation oncologist from Yonsei Cancer Center. All patients were treated by intensity-modulated radiation therapy (IMRT) with a conventional linear accelerator (LINAC) and TomoTherapy. Of the 160 patient scans, 20 scans containing barium-contrast bladder and metal-inserted spine were excluded from this study. The remaining 140 scans were divided into 4 sets for 4-fold cross-validation. Each fold consisted of 105 cases for training and 35 for validating and testing (10 for validating and 25 for testing the trained network).

All PTV CT patients were volumetric datasets in three dimensions, with a median shape of 512 × 512 × 250 and median spacing (0.9766, 0.9766, 2). These datasets were resampled to the same target spacing (2, 2, 2) and embedded into a 256 × 256 × 256 3D volumetric space [35]. After normalizing and window leveling [−200, 250] [36,37,38,39], to enhance the contrast and texture of soft tissue, the foreground of input voxels was selected from the background by an intersection with mask voxels images using MATLAB R2022a. To increase the amount of data for training the network, we augmented the CT images (used for training phases) by rotating them randomly from −0.5 to 0.5 in horizontal, vertical, and axial directions, contrast transforming them randomly from 0.75 to 1.25, and adding noise randomly with a variance that ranged from 0 to 0.1. These data augmentations used BatchGenerators Library provided by the Division of Medical Image Computing of the German Cancer Research Center (DKFZ).

### 2.4. Implementation and Evaluation

The modified VT U-Net v.2 networks were implemented on a personal workstation with dual accelerated GPU (NVIDIA 3090, A6000, Santa Clara, CA, USA), using Python 3.8 (http://www.python.org (accessed on 3 May 2022)) and PyTorch 1.11.1 (http://www.pytorch.org (accessed on 21 May 2022)). The original CT images of 512 × 512 × N voxels had an intensity corresponding to the Hounsfield unit (HU), where N ranged from 61 to 375. During training in the transformer network, the input images were normalized to a range from 0 to 1 [40]. The network was trained using the AdamW optimizer (an open-source Pytorch library) and a modified loss function that combined cross entropy and dice loss under deep supervision. The training ran 1300 epochs, each containing 250 iterations and early stopping. The learning scheduler used PolyLR with a learning rate of 1 × 10^−4^, determined empirically (https://github.com/himashi92/VT-UNet/blob/main/VTUNet/vtunet/training/learning_rate (accessed on 21 May 2022)). Table 2 specifies the network hyper-parameters used in common for both CNN-based and transformer-based networks.

The fully transformer and hybrid networks required the determination of additional hyper-parameters, including the patch size, network architecture, and hyper-parameters, as detailed in Table 3. The hyper-parameters for the existing networks were selected as the values recommended in the published manuscripts. However, the process of hyper-parameters tuning, explained in the subsequent section, led to the selection of the embedded dimension (128) and patch size (128 × 128 × 128) for the modified VT U-Net v.2. The number of heads was adaptively chosen for considering the embedded dimension and the pre-trained model. Meanwhile, the window size followed the Swin-Unetr and VT U-Nets settings and was not optimized.

The proposed transformer architecture, which underwent additional hyper-parameter adaptation on the VT U-Net v.2, was compared to several other networks, including the conventional 3D U-Net [41], nnU-Net, Swin-Unetr, nnFormer, VT U-Net v.1, and VT U-Net v.2. The segmentation accuracy of the proposed network was assessed using the dice similarity coefficient (DSC) and 95% Hausdorff distance (HD95) compared to the other networks. The HD95 was calculated using the 95th percentile of the lengths to minimize a small subset of outliers [19,42].

**Table 2:** Hyper-parameters of convolutional neural network (CNN), hybrid, and transformer-based networks.

Network	Learning Rate	Optimizer	Loss Function	Epoch
3D U-Net [42]	1 × 10^−4^	Adam	Dice + BCE	300
nnU-Net [13,14]	1 × 10^−2^	SGD	Dice + CE + DS	150 (×250)
Swin-Unetr [12]	1 × 10^−4^	AdamW	Dice + CE	400–1000
nnFormer [20]	1 × 10^−2^	SGD	Dice + CE + DS	1300 (×250)
VT U-Net v.1 [21]	1 × 10^−4^	Adam	Dice + BCE	400–500
VT U-Net v.2 [22]	1 × 10^−4^	AdamW	Dice + CE	1300 (×250)
Proposed	1 × 10^−4^	AdamW	Dice + CE + DS	1300 (×250)

**Table 3:** Transformer hyper-parameters of hybrid (CNN and transformer) and transformer-based methods.

Network	Embedded Dimension	Patch Size	Number of Blocks	Window Size	Number of Heads	Parameters
Swin-Unetr [12]	48	96 × 96 × 96	[2, 2, 2, 2]	[7, 7, 7, 7]	[3, 6, 12, 24]	62.8 M
nnFormer [20]	96	128 × 128 × 128	[2, 2, 2, 2]	[4, 4, 8, 4]	[3, 6, 12, 24]	37.7 M
VT U-Net v.1 [21]	96	128 × 128 × 128	[2, 2, 2, 1]	[7, 7, 7, 7]	[3, 6, 12, 24]	20.8 M
VT U-Net v.2 [22]	96	128 × 128 × 128	[2, 2, 2, 1]	[7, 7, 7, 7]	[3, 6, 12, 24]	30.6 M
Proposed	128	128 × 128 × 128	[2, 2, 2, 1]	[7, 7, 7, 7]	[4, 8, 16, 32]	36.7 M

## 3. Results

### 3.1. Quantitative Analysis for PTV Auto-Segmentation

**Figure 4:**
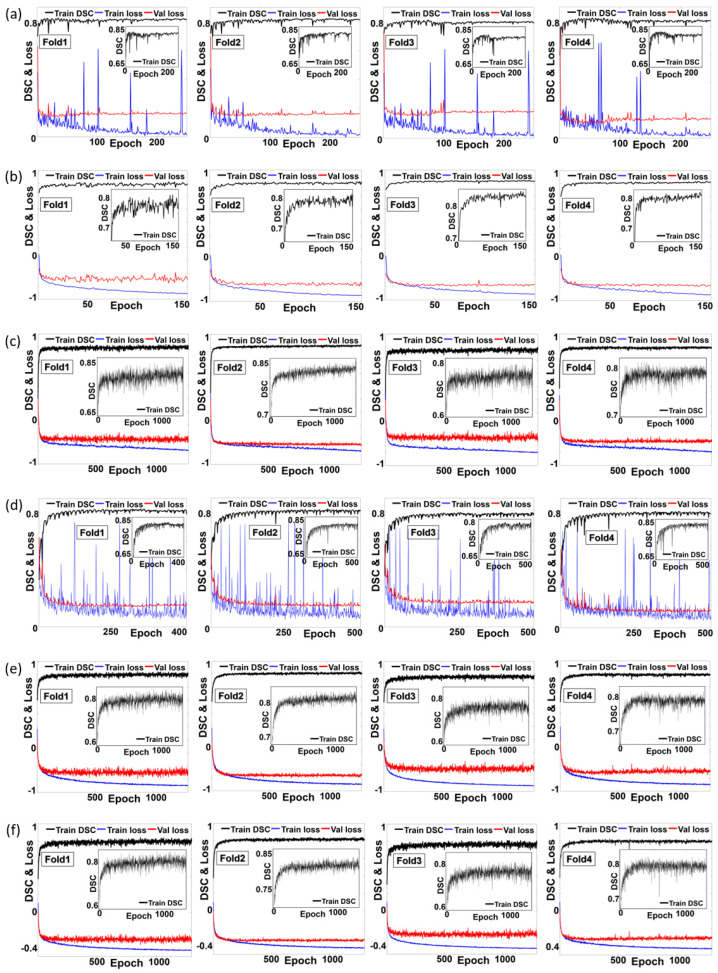
(**a**) Training and validating loss and accuracy for the investigated networks: (**a**) 3D U-Net, (**b**) nnU-Net, (**c**) nnFormer, (**d**) VT U-Net v.1, (**e**) VT U-Net v.2, and (**f**) Proposed. (Swin-Unetr was excluded as it recorded validating accuracy only).

Figure 4 reveals the training and validating loss and accuracy for all investigated networks in this study. By illustrating the pattern of losses and DSCs (accuracy) in training and validating phases over the number of epochs, it showed that training and validating of the investigated convolution-based and transformer-based networks were appropriately implemented. Table 4 shows the quantitative analysis of the segmentation accuracy of the networks used for the whole pelvis PTV segmentation for prostate cancer patients. On average, the modified VT U-Net v.2 outperformed the other networks in terms of DSC and HD95 across the four-fold cross-validation. The unmodified VT U-Net v.2 was expected to perform similarly to its modification in each fold. However, some slight differences arose due to additional hyper-parameter adjustment (patch size and embedded dimension) and the adoption of deep supervision in the loss function. Swin-Unetr, which combined CNN and transformer, showed competitive performance against the proposed network. It is worth noting that VT U-Net v.1, which consisted of the fully transformer network architecture, resulted in poor segmentation accuracy. Meanwhile, nnU-Net occasionally displayed comparable performance to the proposed network, despite being based on convolutional blocks. Consequentially, it implied that the suitable hyper-parameter selection for the specific network architecture would be important, as did the type of network architecture for auto-segmentation.

**Figure 5:**
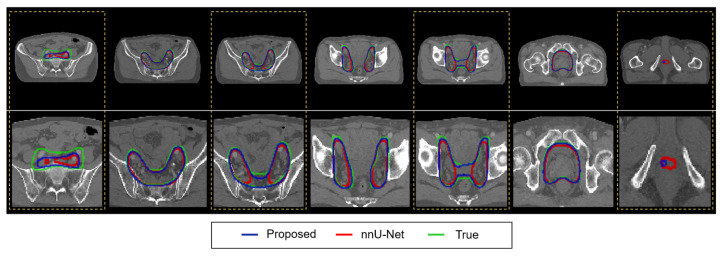
Qualitative analysis of the modified VT U-Net v.2 and nnU-Net for PTV auto-segmentation. The top, bottom, and joint in PTV made a difference in performance in both models (yellow). (Upper row: original images, bottom row: enlarged view).

Table 5 presents the statistical analysis of the proposed network compared to other networks for the 25 testing cases in each fold and the total 100 cases in the combined fold 1 to 4, represented by the *p*-value. In all folds combined, the differences between the proposed network and other networks were statistically significant (*p* < 0.05) in most cases for DSC and HD95, except for HD95 against nnU-Net, Swin-Unetr, and VT U-Net v.2. In the fold-specific comparison, the nnU-Net and VT U-Net v.2 were highly competitive with the proposed network. VT U-Net v.2, the origin of the proposed network, showed comparable results, possibly due to the relatively small sample size. Meanwhile, the nnU-Net demonstrated the effectiveness of the hyper-parameter optimization in statistical analysis and the averaged outcomes. In a single testing case, Figure 5 illustrates the segmented contours of the whole pelvis prostate PTV from the modified VT U-Net v.2 and nnU-Net, along with the ground truth. It turned out that the two networks had similar performance, while the difference was found in the transition area from the lymphatic nodes to the prostate tumor bed, as highlighted by the dotted yellow.

### 3.2. Hyper-Parameter Tuning

In investigating the impact of hyper-parameter selection, the patch size varied from 96 × 96 × 96 through 128 × 128 × 128 to 160 × 160 × 160 on the modified VT U-Net v.2. The embedded dimension was also carefully chosen with options 96, 128, and 192, which corresponded to the setting of the pre-trained networks. In addition, as stated in the preceding section, we adaptively tuned the head numbers regarding the embedded dimension, and the window size was seven, as in the previous transformer-based networks.

**Figure 6:**
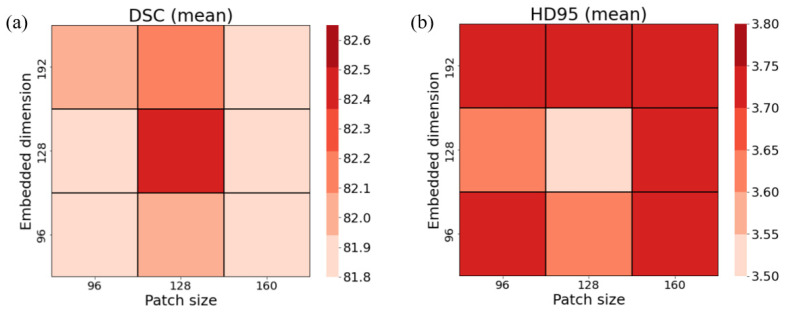
Hyper-parameter selection in the proposed network (the modified VT U-Net v.2). (**a**) DSC and (**b**) HD95 across nine combinations of the embedded dimension (head numbers) and patch size.4. Discussion.

Table 6 presents the DSCs and HD95s for various combinations of patch size and embedded dimension, which were computed over four different folds. Although there were some exceptional cases, the proposed network achieved the largest DSC and the lowest HD95 when the patch size was 128 × 128 × 128, and the embedded dimension was 128. It was also found to have a trend that a combination of the large patch size (160 × 160 × 160) of the input for the network and the smaller embedded dimension (96 or 128) or vice versa yielded greater segmentation accuracy. When averaging DSCs and HD95s for the testing cases belonging to folds 1 through 4, the selected combination of patch size of 128 for three dimensions and the embedded dimension of 128 seemed more explicit against the other possible combinations shown in Figure 6. Moreover, there was a slight indication that the patch size of 128 was a stronger constraint for enhancing the segmentation accuracy relative to the embedded dimension. Table 7 lists the *p*-values following the statistical analysis between the selected combination and the others. In most cases, the selected combination had statistically significant differences against the combinations in a consistently exceptional case for the DSC and HD95 criterion. In the statistical analysis, however, it was difficult to discern which network hyper-parameter affected the segmentation accuracy the most.

This work was motivated by a hypothesis that the limited application of CNN to auto-segmentation of PTV may be associated with the inherent characteristics of CNN-based networks. Specifically, these networks propagated local imaging features throughout the depth of layers, which could limit their ability to capture global imaging information. Therefore, the vision transformer has attempted the architectural transformation as an alternative, which can bring in more global imaging information by shifting the regional patches to the original patch during network training. Out of several variants in transformer architecture, the VT U-Net v.1 with fully transformer layers was chosen as a candidate for auto-segmenting the whole pelvis PTV to investigate the effectiveness of the architectural transformation. Additionally, it was well known that the other approach regarding the hyper-parameter selection was introduced under the name of nnU-Net. This approach aimed to identify the suitable combinations of hyper-parameters by considering the GPU computational efficiency and the capacity based on their statistics, along with the slightly different loss definition called deep supervision. Although the updated VT U-Net (v.2) embraced some critical features of nnU-Net, especially in computational efficiency, it may require further modifications to take transformer-oriented hyper-parameters into account. Thus, this work did not adopt the given hyper-parameters, but instead adjusted vital parameters such as the patch size and embedded dimension (associated with head numbers) to find a suitable combination that can enhance the segmentation accuracy.

As a result of the extensive investigations and comparisons against the existing networks, the modified VT U-Net v.2 yielded the highest average DSC and lowest average HD95 quantitatively, from the four-fold cross-validations, followed by VT U-Net v.2, nnU-Net, Swin-Unetr, nnFormer, VT U-Net v.1, and 3D U-Net. Concerning the statistical analysis, the VT U-Net v.2 and nnU-Net turned out to be the most competitive models. Unexpectedly, VT U-Net v.1 resulted in poor accuracy despite the fully transformer architecture applied, which might have been derived from two reasons. First, the nnU-Net, well-customized to the CNN-based framework, did not lose its competitiveness in the PTV auto-segmentation relative to the transformer-based architecture. The auto-segmentation results showed remarkable differences between VT U-Net v.1 and v.2 even though they shared the same network architecture, which implied that considering the network hyper-parameters should be significant. We found that there were two key parameters that highly affected the PTV segmentation accuracy, the patch size and embedded dimension. Hence, we trained the proposed network while varying those two important network hyper-parameters under nine different conditions. It involved 36 training sessions for the 4-fold cross-validation, each lasting 4.2 days. From our observation, the variation of two influential network hyper-parameters, patch size and embedded dimension, led to non-negligible differences in the segmentation accuracy, as seen in quantitative results and statistical analysis of Table 6 and Table 7. Importantly, the VT U-Net v.2 chose the embedded dimension to be 96 as a default, while the embedded dimension of 128 turned out to attain greater PTV segmentation accuracy. Along with them, the patch size of 128 × 128 × 128 yielded the best results.

The dataset used in this work consisted of 140 CT scans with a prior on the whole pelvis prostate PTV given. Although the number of CT scans was sufficiently large for the network training and evaluation, the proposed network was assessed using only a single case, which did not fully generalize the selected network hyper-parameters to be optimal for other clinical sites. However, it is well known that the planning CT images used for radiotherapy had similar image sizes (512 × 512 with 100–200 slices) and image resolutions (0.97~1 mm in x- and y-axis and 2~3 mm in z-axis). Considering the fact that the hyper-parameters focused on in this work were highly involved in the resolution and size of input image, the hyper-parameter posed in this work would work out properly for the other datasets unless the PTV size were too much small. Another point for discussion is the resolution of hyper-parameters considered in this work, such as the embedded dimensions of 96, 128, and 192 and patch sizes of 96, 128, and 160. The values were chosen based on the specifications of the pre-trained models used, as the VT U-Net was based on the pre-trained model. There could be slightly different combinations of those parameters with denser sampling. Considering the network architecture consisting of down- and up-sampling, the possible values are somewhat constrained to 64, 96, 128, 160, and 192, etc., in the given hardware. The results found in this work might still be validated in this sense. Finally, the hyper-parameter tuning studied in this work could only partially comprehend some network hyper-parameters in the transformer. As shown in Table 3, the head sizes were adaptively changed from [3, 6, 12, 24] for each layer to the enlarged extent depending on the embedded dimension, [4, 8, 16, 32] for the embedded dimension of 128. The window size was fixed to seven for each layer as most existing transformers selected the value. The previous work, nnFormer, attempted to adopt a variable window size across the network layers [4, 4, 8, 4] instead of the fixed number seven. Still, it did not provide improved accuracy when applied to the proposed network (DSC of 81.9 and HD95 of 3.6). This work focused on the hyper-parameters associated with the size of the first features entering the vision transformer. In the long run, further extensive investigations are required to determine the optimal parameter selections regarding network architectures. Although there may be a long journey in the hyper-parameter optimizer for the new network architecture, vision transformer, the primary findings and insights discussed in this work are a major milestone in emphasizing the hyper-parameter setting for PTV auto-segmentation using the transformer-based networks.

## 4. Conclusions

This work proposed a fully transformer-based network to auto-segment the whole pelvis PTV for prostate cancer patients with appropriate hyper-parameter selection. It successfully demonstrated that the network transformation from the CNN-based to the transformer-based approach and the choice of essential hyper-parameters oriented to the transformer are important to enhance the segmentation accuracy. Additionally, our proposed network with 128 embedded layers and 128 × 128 × 128 patch size led to a promising performance compared to other investigated networks (CNN, hybrid, and transformer-based networks), with an average DSC of 82.5 and HD95 of 3.5 for 4-fold cross-validation.

## Figures and Tables

**Table 1:** Functions regarding network hyper-parameter selection. “o” denotes the automatic implementation of the function, while the semi-automatic in “△”. “—” indicates not applicable in the model, while “×” is the disuse despite the benefit of the function.

	nnU-Net	VT U-Net v.1	VT U-Net v.2	Modified VT U-Net v.2 (Proposed)
Pre- and post-processing and loss				
Oversampling ^1^	o	×	o	o
Post-processing ^2^	o	×	o	o
Deep supervision	o	×	×	o
Hyper-parameter & architecture				
Batch size	o	×	o	o
Patch size	o	×	×	o
Embedded dimension	—	×	×	o
Head number	—	×	×	△ (adaptive to embedded dimension)
Window size	—	×	×	×
Architecture	o	×	△	o (same as nnU-Net)

Oversampling ^1^: sampling patches centering a voxel chosen randomly in the target volume with ratio. Post-processing ^2^: removing all but the largest connected foreground region.

**Table 4:** Segmentation results of CNN, hybrid, and transformer architectures on the PTV dataset. ↑ means higher is better. The best results are bolded while the second best are underlined, and experimental results of baselines were acquired from 3D U-Net. All experiments were run as 4-fold cross-validation. “↓” means lower is better.

Method	Dice Similarity Coefficient (DSC) ↑					95% Hausdorff Distance (HD95) ↓				
	Fold1	Fold2	Fold3	Fold4	Mean	Fold1	Fold2	Fold3	Fold4	Mean
3D U-Net	80.96	77.98	76.46	76.75	78.0 ***	3.23	4.19	5.55	4.45	4.4 ***
nnU-Net	83.95	82.02	79.25	82.44	81.9 *	2.70	3.60	5.14	2.94	3.6
Swin-Unetr	82.68	81.87	79.47	**83.06**	81.8 *	3.00	3.65	**4.97**	**2.75**	3.6
nnFormer	83.31	81.31	79.85	82.39	81.7 **	2.80	3.78	5.13	3.12	3.7 *
VT U-Net v.1	80.21	76.65	75.08	76.25	77.0 ***	3.35	4.34	5.44	3.93	4.3 ***
VT U-Net v.2	84.12	82.30	79.82	82.61	82.2 **	2.72	3.60	5.04	3.01	3.6
Proposed	**84.20**	**82.65**	**80.13**	82.82	**82.5**	**2.49**	**3.52**	4.98	3.01	3.5

*: *p*-value < 0.05, **: *p*-value < 0.01, ***: *p*-value < 0.001.

**Table 5:** Statistical analysis of CNN, hybrid, and transformer architectures. The *p*-value of the models <0.05 indicates that the performance difference is statistically significant.

Method	DSC					HD95				
	Fold1	Fold2	Fold3	Fold4	All folds	Fold1	Fold2	Fold3	Fold4	All Folds
3D U-Net	**<0.001**	**<0.001**	**<0.001**	**<0.001**	**<0.001**	**0.001**	**<0.001**	**0.004**	**<0.001**	**<0.001**
nnU-Net	0.410	0.126	0.255	0.206	**0.040**	0.070	0.473	0.122	0.479	0.080
Swin-Unetr	**0.022**	**0.020**	**0.030**	0.339	**0.020**	**0.007**	0.123	0.091	0.392	0.210
nnFormer	**0.046**	**0.013**	0.389	0.122	**0.010**	**0.028**	0.116	0.468	0.181	**0.020**
VT U-Net v.1	**<0.001**	**<0.001**	**<0.001**	**<0.001**	**<0.001**	**<0.001**	**<0.001**	0.050	**<0.001**	**<0.001**
VT U-Net v.2	0.331	0.051	0.117	0.106	**0.010**	0.426	0.448	0.170	0.480	0.090

**Table 6:** Hyper-parameter selection in the proposed network (the modified VT U-Net v.2). DSC and HD95 were measured 4-fold.

Hyper-Parameter		DSC ↑				HD95 ↓			
Patch Size	Embedded Dimension	Fold1	Fold2	Fold3	Fold4	Fold1	Fold2	Fold3	Fold4
96	96	83.57	82.09	79.57	82.33	2.72	3.78	5.02	3.12
96	128	83.78	82.34	79.35	82.25	2.52	3.60	4.98	3.12
96	192	**84.36**	82.05	79.52	81.98	2.68	4.03	5.02	3.22
128	96	83.72	82.32	79.77	82.39	2.73	3.70	5.05	3.07
128	128	84.20	**82.65**	80.13	**82.82**	**2.49**	**3.52**	4.98	**3.01**
128	192	83.96	81.89	**80.45**	82.23	2.63	3.78	**4.94**	3.44
160	96	84.16	81.87	79.37	81.87	2.78	3.59	5.07	3.26
160	128	83.65	82.14	79.65	82.11	2.64	3.80	5.02	3.19
160	192	84.03	81.54	79.85	81.88	2.61	3.78	4.96	3.25

↑ means higher is better, while ↓ means lower is better. The best results are bolded while the second best are underlined.

**Table 7:** Statistical analysis (*p*-value) of hyper-parameter selection in the proposed network. The *p*-value of the models <0.05 indicates that the performance difference is statistically significant.

Hyper-Parameter		DSC					HD95				
Patch Size	Embedded Dimension	Fold1	Fold2	Fold3	Fold4	All Folds	Fold1	Fold2	Fold3	Fold4	All Folds
96	96	0.111	**0.018**	0.061	**0.043**	**0.001**	**0.021**	0.157	0.149	0.190	**0.006**
96	128	0.185	0.144	**0.043**	0.069	**0.005**	0.087	0.108	0.475	0.405	0.079
96	192	0.365	0.087	0.072	**0.005**	**0.009**	0.179	0.199	0.380	0.081	**0.026**
128	96	0.101	0.124	**0.021**	**0.046**	**0.004**	**0.026**	0.236	0.222	0.166	**0.019**
128	192	0.193	0.118	0.089	**0.020**	0.059	0.089	0.145	0.301	**0.021**	**0.018**
160	96	0.451	0.154	**0.008**	**0.001**	**<0.001**	0.058	0.176	0.076	**0.005**	**<0.001**
160	128	0.137	0.066	0.127	**0.007**	**0.002**	0.228	0.080	0.389	**0.007**	**0.006**
160	192	0.347	**0.023**	0.173	**<0.001**	**0.001**	0.294	**0.036**	0.172	**0.003**	**0.020**

The *p*-value < 0.05 are bolded.

## Data Availability

The datasets generated during the current study will be available from the corresponding author upon reasonable request.
